# Cervical Spondylosis Mimicking Meningitis in a Patient With High BMI: A Case Report

**DOI:** 10.7759/cureus.106273

**Published:** 2026-04-01

**Authors:** Nidhi Kakkar

**Affiliations:** 1 Department of Medicine for Older People, Stockport NHS Foundation Trust, Stockport, GBR

**Keywords:** bacterial meningitis, cervical disc herniation, cervical spine disorders, lumbar puncture (lp), procalcitonin

## Abstract

This report describes the case of a 58-year-old female patient with a history of hypertension, dyslipidemia, and childhood asthma, who presented with neck stiffness, headache, and mild fever, raising concern for meningitis. Despite her high BMI and reluctance to undergo a lumbar puncture, she was initially treated with intravenous aciclovir and ceftriaxone while microbiological tests, including procalcitonin, ruled out bacterial infection. Laboratory markers, such as CRP and white blood cell count, improved over time, and cervical spine X-rays revealed cervical spondylosis with C4-C5 protrusion, explaining her symptoms. As a result, antimicrobial therapy was discontinued, and she was referred to a local spine specialist. This case underscores the need to consider cervical spine pathology as a differential diagnosis in patients with meningitis-like symptoms, particularly when invasive investigations are not feasible.

## Introduction

Neck stiffness, headache, and fever are classical signs that often raise suspicion for meningitis, a potentially life-threatening condition requiring prompt diagnosis and treatment [1]. Lumbar puncture remains the gold standard for confirming meningitis, but invasive procedures can be technically challenging or contraindicated in certain patient populations, such as those with obesity, bleeding disorders, or patient refusal. Misattributing symptoms to infectious causes may lead to unnecessary antimicrobial therapy, prolonged hospitalization, and procedural complications.

Cervical spondylosis is a common degenerative disorder of the cervical spine, particularly in older adults. It is characterized by disc degeneration, osteophyte formation, and potential nerve or spinal cord compression, which can manifest as neck stiffness, localized pain, and sometimes mild systemic symptoms, including low-grade fever [2]. Although rare, cervical spondylosis can mimic the clinical presentation of meningitis, complicating the diagnostic process.

Previous reports have highlighted cases where cervical spine pathology led to symptoms suggestive of meningeal irritation, underscoring the need for careful differential diagnosis. Biomarkers such as procalcitonin may aid in ruling out bacterial infections, while imaging studies provide definitive structural assessment [3]. This case illustrates the clinical challenge of distinguishing cervical spondylosis from meningitis, emphasizing the importance of a systematic evaluation that integrates clinical findings, laboratory markers, and imaging studies to guide management.

## Case presentation

Patient description and case history

A 58-year-old female, weighing 96 kg with a body mass index of 33 kg/m², presented to the emergency department with a two- to three-day history of neck stiffness, rigidity, headache, and low-grade fever. She had a past medical history of hypertension, hyperlipidemia, and childhood asthma. The patient reported progressive neck stiffness primarily in the posterior cervical region, associated with restricted motion and intermittent discomfort radiating to the shoulders. There were no neurological symptoms such as numbness, weakness, or paresthesia, and she denied photophobia, nausea, vomiting, or any changes in consciousness. The patient expressed apprehension regarding lumbar puncture due to her elevated BMI and personal concern about procedural risk.

Physical examination

On admission, vital signs were the following: temperature 37.8°C, blood pressure 138/82 mmHg, heart rate 88 beats per minute, respiratory rate 16 breaths per minute, and oxygen saturation of 98% on room air. Neurological examination showed the patient was alert and oriented, with intact cranial nerves, normal motor strength (5/5 in all extremities), preserved reflexes, and no sensory deficits. Neck examination revealed significant stiffness and mild tenderness localized over the C4-C5 region, with limited flexion and rotation. There were no signs of meningism, including Brudzinski’s or Kernig’s signs. Cardiovascular, respiratory, and abdominal examinations were unremarkable.

Pathological and laboratory investigations

Initial laboratory investigations are summarized in Table 1. White blood cell count and CRP were mildly elevated initially but trended downward over serial measurements. Procalcitonin was <0.05 ng/mL, effectively ruling out bacterial infection. Blood cultures were negative. Liver and renal function tests were within normal limits.

**Table 1:** Serial laboratory investigations, including full blood count, urea and electrolytes, C-reactive protein, and procalcitonin over the course of hospitalization. Day 1 represents admission values, with follow-up measurements on Day 3 and Day 5. Values are reported as follows: white blood cell (WBC) count (measured in ×10⁹ cells per liter (×10⁹/L)), neutrophils and lymphocytes (percentage of total WBCs (%), hemoglobin (grams per deciliter (g/dL)), platelets (×10⁹ platelets per liter (×10⁹/L)), urea (millimoles per liter (mmol/L)), creatinine (micromoles per liter (µmol/L)), sodium (millimoles per liter (mmol/L)), potassium (millimoles per liter (mmol/L)), chloride (millimoles per liter (mmol/L)), bicarbonate (millimoles per liter (mmol/L)), C-reactive protein (milligrams per liter (mg/L)), and procalcitonin (nanograms per milliliter (ng/mL)). Reference ranges are provided for comparison. Trends demonstrate normalization of inflammatory markers and stable renal function, supporting a non-infectious etiology for the patient’s symptoms.

Parameter	Day 1	Day 3	Day 5	Reference Range
Full Blood Count				
White blood cell count (×10⁹/L)	9	8.5	8.2	4–10
Neutrophils (%)	72	68	65	40–70
Lymphocytes (%)	20	22	25	20–45
Hemoglobin (g/dL)	13.5	13.4	13.3	12–16
Platelets (×10⁹/L)	250	245	240	150–400
Urea & Electrolytes				
Urea (mmol/L)	5.2	5	4.8	2.5–7.1
Creatinine (µmol/L)	78	76	74	45–90
Sodium (mmol/L)	138	137	137	135–145
Potassium (mmol/L)	4.2	4	4.1	3.5–5.0
Chloride (mmol/L)	102	101	101	95–110
Bicarbonate (mmol/L)	24	24	25	22–28
Inflammatory Markers				
C-reactive Protein mg/L)	18	14	12	0–5
Procalcitonin (ng/mL)	0.05	0.04	0.03	<0.1

**Figure 1:**
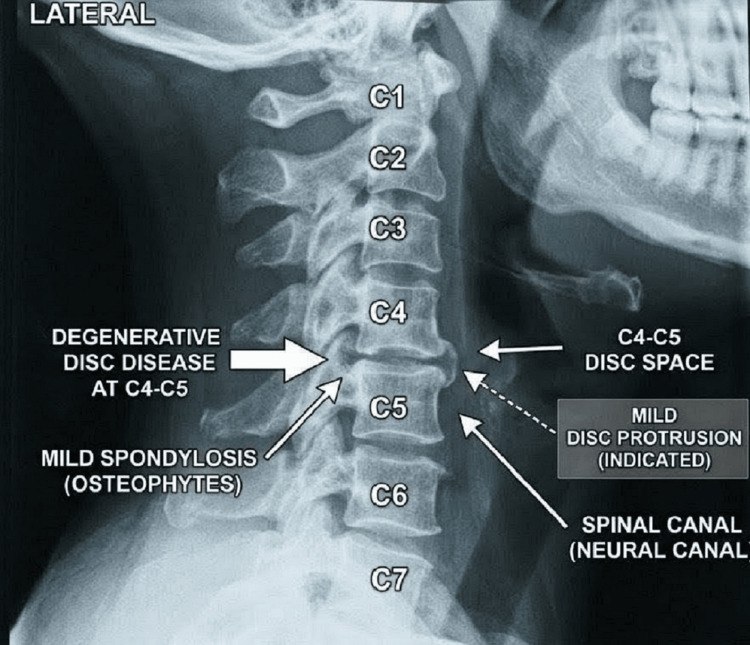
Cervical spine X-ray, lateral view, demonstrated changes at the C4-C5 level with disc protrusion and mild spondylosis.

The cervical spine X-ray demonstrated degenerative changes at the C4-C5 level with disc protrusion and mild spondylosis (Figure 1). No acute fracture or spinal instability was observed.

Treatment plan

Given initial suspicion for viral or bacterial meningitis, empirical intravenous therapy with aciclovir and ceftriaxone was initiated. Microbiology consultation recommended serial monitoring of inflammatory markers and procalcitonin to guide antibiotic stewardship. Once laboratory findings and imaging suggested cervical spondylosis as the cause of her symptoms, antiviral therapy was discontinued after five days, and ceftriaxone after seven days [4]. Pain management was provided with analgesics, and the patient was referred to physiotherapy and the outpatient spine team for long-term management.

Expected outcome

Based on the initial clinical presentation and differential diagnosis, the expected outcome was cautious. If the symptoms were infectious in origin, prompt treatment would prevent neurological deterioration; if cervical spine pathology was responsible, conservative management and rehabilitation were expected to relieve neck stiffness and prevent recurrent episodes.

Actual outcome

The patient’s neck stiffness and headache gradually improved with analgesics and physiotherapy. No infectious complications were observed, and she remained afebrile. On discharge, she was referred to the local spine service for outpatient management and ongoing monitoring. At follow-up, the patient reported improved cervical mobility and resolution of headache, consistent with the diagnosis of cervical spondylosis rather than meningitis.

## Discussion

Meningitis, especially when accompanied by neck stiffness, fever, and headache, remains a medical emergency, requiring prompt diagnosis and treatment. The classical presentation of these symptoms often leads to suspicion of meningitis, with lumbar puncture being the gold standard for diagnosis. However, as illustrated by this case, not all patients with these symptoms have an infectious aetiology. Cervical spondylosis, a common degenerative spinal disorder, can also present with neck stiffness, headache, and even mild systemic symptoms, sometimes mimicking meningitis. This overlap in symptoms can pose a diagnostic challenge and requires a careful and systematic approach.

Cervical spondylosis and meningitis-like symptoms

Cervical spondylosis is frequently encountered in older adults and is characterized by degenerative changes in the cervical spine, such as disc protrusions and osteophyte formation. It often presents with localized neck pain, stiffness, and sometimes radicular symptoms, including discomfort radiating to the shoulders or upper limbs. While systemic symptoms like low-grade fever are less commonly reported, they can occur in cases where there is significant inflammation or nerve root irritation [5]. The presence of neck stiffness and headache in such cases can easily be mistaken for meningitis, especially when other classical signs of meningism, such as photophobia and altered consciousness, are absent, as was the case with our patient.

Previous studies have highlighted the diagnostic confusion between cervical spine pathology and meningitis, especially in patients without obvious neurological deficits. Tracy and Bartleson (2010) discussed how cervical spondylosis can result in clinical manifestations that overlap with those of meningitis, such as neck stiffness and headache, but also noted that more severe neurological deficits, like myelopathy, are usually present in advanced cases [4]. Similarly, Bakhsheshian et al. (2017) emphasized that cervical spondylosis could lead to symptoms that mimic both bacterial meningitis and viral infections, complicating the diagnostic process, particularly when the physical examination is inconclusive and lumbar puncture is contraindicated or refused [3].

The role of procalcitonin and other biomarkers

Biomarkers such as procalcitonin have proven helpful in distinguishing between bacterial infections and other causes of systemic inflammation [5]. In the present case, a negative procalcitonin result was critical in ruling out bacterial meningitis, preventing unnecessary antibiotic treatment and guiding more targeted management [6]. Studies by Schuetz et al. (2009) and Brouwer et al. (2010) have shown that procalcitonin is a valuable marker in diagnosing bacterial infections and improving antibiotic stewardship, especially when the clinical presentation is ambiguous, as in our patient [6, 7].

The use of procalcitonin, in conjunction with imaging studies, such as cervical spine X-rays or MRI, can effectively differentiate between infectious and non-infectious causes of neck stiffness. This approach has been supported by McIntire and Green (2001), who found that procalcitonin levels were often low in patients with non-infectious conditions, even when presenting with symptoms suggestive of meningitis [2]. This aligns with the findings in our case, where serial procalcitonin levels were consistently low, and imaging revealed cervical spondylosis rather than an infectious cause.

Imaging studies and early diagnosis

The role of imaging, particularly cervical spine X-rays or MRI, is crucial in cases where the clinical presentation is suggestive of both meningitis and cervical spine pathology [8]. The literature suggests that imaging studies should be considered when the clinical diagnosis is uncertain or if invasive procedures such as lumbar puncture are not feasible. McIntire and Green (2001) recommended cervical spine imaging as part of the differential diagnosis in patients with meningitis-like symptoms, especially when there is a history of degenerative spinal disease or risk factors for cervical spine pathology [2].

Moreover, studies have shown that early imaging can prevent the unnecessary use of antibiotics and invasive procedures, such as lumbar puncture, which may have complications in patients with certain risk factors, such as obesity or anatomical challenges. This approach is endorsed by Carter and McGill (2022), who emphasized the importance of an integrated diagnostic strategy combining clinical, laboratory, and imaging findings in managing suspected meningitis cases [1].

Challenges in patients with high BMI

In patients with a high BMI, performing a lumbar puncture can be technically challenging, and there may be an increased perception of procedural risk, as demonstrated in this case. Studies such as those by Tracy and Bartleson (2010) have noted that obesity can complicate the assessment of meningitis due to difficulties in performing procedures like lumbar puncture and the interpretation of physical exam findings [4]. Additionally, obesity may increase patient anxiety and reluctance to undergo invasive diagnostic procedures. These factors further underline the importance of considering non-infectious causes, such as cervical spondylosis, especially in patients with risk factors for spinal degeneration or a history of neck pain [9].

Prevention of unnecessary treatment and antibiotic stewardship

This case highlights the importance of preventing unnecessary antimicrobial therapy in patients with non-infectious causes of meningitis-like symptoms. The early consideration of alternative diagnoses, such as cervical spine pathology, coupled with the use of diagnostic tools like procalcitonin and imaging studies, can reduce the unnecessary use of antibiotics [10]. The concept of antibiotic stewardship is becoming increasingly vital in the management of suspected infections, as overuse of antibiotics contributes to resistance and other complications. Brouwer et al. (2010) discussed how early identification of non-infectious causes, including cervical spondylosis, can prevent the misapplication of antibiotics and reduce the overall healthcare burden [6].

## Conclusions

Cervical spondylosis can present with symptoms that mimic meningitis, including neck stiffness, headache, and mild fever. In patients who are apprehensive about invasive procedures, early imaging and laboratory markers such as procalcitonin can help rule out infection and guide appropriate management. Recognizing non-infectious causes of meningitis-like presentations can prevent unnecessary antibiotic exposure and facilitate targeted care.
